# Inorganic Skeleton Reinforcement—A Generic Approach to Improve the Mechanical Properties of Biochar

**DOI:** 10.3390/nano13081298

**Published:** 2023-04-07

**Authors:** Zhikai Chen, Xiaoli Jiang, Yagang Zhang, Wei Li, Zhiqiang Tang, Yanxia Liu, Lin Zhao

**Affiliations:** 1 School of Materials and Energy, University of Electronic Science and Technology of China, Chengdu 611731, China; 2 State Key Laboratory of Electronic Thin Films and Integrated Devices, University of Electronic Science and Technology of China, Chengdu 611731, China

**Keywords:** inorganic skeleton, mechanic properties, biochar, adsorption performance

## Abstract

Biochar is considered as a promising candidate for emerging sustainable energy systems and environmental technology applications. However, the improvement of mechanical properties remains challenges. Herein, we propose a generic strategy to enhance the mechanical properties of bio-based carbon materials through inorganic skeleton reinforcement. As a proof-of-concept, silane, geopolymer, and inorganic gel are selected as precursors. The composites’ structures are characterized and an inorganic skeleton reinforcement mechanism is elucidated. Specifically, two types of reinforcement of the silicon-oxygen skeleton network formed in situ with biomass pyrolysis and the silica-oxy-al-oxy network are constructed to improve the mechanical properties. A significant improvement in mechanical strength was achieved for bio-based carbon materials. The compressive strength of well-balanced porous carbon materials modified by silane can reach up to 88.9 kPa, geopolymer-modified carbon material exhibits an enhanced compressive strength of 36.8 kPa, and that of inorganic-gel-polymer-modified carbon material is 124.6 kPa. Moreover, the prepared carbon materials with enhanced mechanical properties show excellent adsorption performance and high reusability for organic pollutant model compound methylene blue dye. This work demonstrates a promising and universal strategy for enhancing the mechanical properties of biomass-derived porous carbon materials.

## 1. Introduction

Utilizing non-food biomass to produce renewable energy and bio-based materials is a sustainable strategy for addressing environmental deterioration and achieving carbon neutrality. Biochar, a solid material with a porous structure and high carbon content, can be obtained by removing volatile components from biomass feedstocks through pyrolysis in an oxygen-limited or oxygen-free environment [1,2,3]. Compared with fossil-fuel-derived activated carbon and carbon black, biomass pyrolysis to produce biochar is an energy-conserving and sustainable process that helps to reduce anthropogenic CO_2_ emissions [4]. Owing to the advantages of a large specific surface area (1000–2500 m^2^/g), high-cost performance, rich porosity (0.8–0.9 m^3^/g), and rich surface functional groups (-COOH, -CHO, and so on), carbon-based materials extracted from biomass have been used in various fields such as electrocatalysis, energy storage, and environmental remediation [5,6,7,8,9,10]. The main reasons for selecting biomass as the precursor for carbon materials are as follows: (i) Biomass has Earth-abundant resources mainly composed of cellulose, protein, carbohydrates, and a few inorganic minerals, which can provide in situ doping of heteroatoms (such as N, S, and P) for carbon materials. The combination of heteroatoms and carbon materials is crucial for regulating the surface electrochemical and chemical states. Moreover, co-doping can improve the overall performance of the material thanks to the synergistic effect of heteroatoms [11,12,13,14]. (ii) Biomass-derived biochar has a porous skeleton and is formed by overlapping two-dimensional (2D) carbon nanosheets. Two-dimensional carbon materials are widely used in energy systems and environmental technology fields because of their large specific surface area, additional electrochemical active sites, and high level of mechanical flexibility [15,16,17,18,19,20]. However, in the specific application of carbon materials, there are still various problems in use [21,22,23,24,25,26,27]. For example, at present, the process for producing vinyl chloride monomer (VCM) adopts fixed bed reaction technology, which is simple and mature. However, the mechanic properties of the catalyst are poor and it is easy to pulverize, leading to large consumption and low heat transfer efficiency, which limits the catalytic performance of the catalyst. In addition, the poor mechanical strength of biochar materials is unfavorable in terms of transportation, handling, and storage [28].

**Figure 1:**
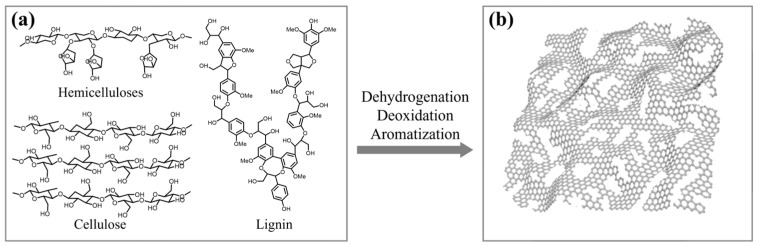
Dehydrogenation, deoxygenation, and graphitization of biomass. (**a**) The chemical structures of hemicelluloses, cellulose, and lignin; (**b**) the actual two-dimensional plane structure of biochar with defects.

Biomass is mainly composed of hemicelluloses, cellulose, and lignin, and these components are pyrolyzed through different pathways and at different rates (Figure 1a). The preparation process of biochar is the graphitization process of dehydrogenation and deoxidation of biomass (Figure 1) [29]. Specifically, the preparation of biomass-based carbon materials through pyrolysis can be divided into three stages. The first stage is the dehydration process, which is accompanied by the partial breakage of chemical bonds. The second stage is the evaporation process, during which volatile organic compounds inside the biomass are released. The third stage is carbonization, which refers to the enrichment of carbon elements and the formation of a relatively stable carbon skeleton structure. Ideally, the structure of obtained biomass-based carbon materials is a fully graphitized two-dimensional planar structure. However, owing to the chain breaking of the biomass precursor and the incomplete graphitization process, the actual microstructure of obtained biomass-based carbon materials is an irregular graphene-like structure with many defects. The irregular structure is composed of multiple benzene rings, condensed aromatic rings, and branched structures. At the molecular level, the excellent mechanical properties of the biomass precursor come from the natural interweaving of biopolymer fibers and tightly packed bulk materials. However, after pyrolysis and carbonization, a large number of defects are formed owing to bond breaking and chain breaking in the carbonization process, which greatly reduces the mechanical properties of biomass-based carbon materials. Specifically, only a small part of the carbon skeleton in the biomass-based carbon material exhibits regular circular graphene structures, and most of it is filled with defects and pores, resulting in an irregular and incomplete structure. Therefore, biomass-based carbon materials are prone to collapse under even small external forces [30,31,32,33,34]. The application of biochar functional materials has been the focus of researchers, while the basic but challenging mechanical properties of biochar have been neglected.

To improve the mechanical properties of biomass-based carbon materials, in this work, mechanically enhanced biochar was prepared by adding an inorganic skeleton reinforcement of tetraethoxysilane (TEOS), fly ash, and cement. There are two technical routes: (1) the inorganic skeleton can be formed in situ by hydrolysis and condensation of TEOS in the process of biochar preparation; (2) the bio-based carbon material is mixed with inorganic skeleton precursors (fly ash, and cement), which provide an inorganic skeleton through the hydration of cement and alkaline activation of fly ash to which bio-based carbon material can attach. There are benefits arising from the formation of the inorganic skeleton. The formed inorganic skeleton provides effective support for delicate and fragile biochar, thus improving the mechanical properties of the bio-based carbon materials. This provides an alternative approach to enhance the mechanical properties of biochar. 

## 2. Materials and Methods

### 2.1. Materials

The ginkgo leaves were collected from the campus of University of Electronic Science and Technology of China. These leaves were cleaned, dried, and ground into ginkgo leaf powder for later use. Ethanol and hydrochloric acid were used as analytical reagents; the carbon powder used was 200-mesh wooden activated carbon. The chemical reagents used in the experiment were purchased from Aladdin Bio-Chem Technology Co., Ltd. (Shanghai, China) TEOS (Si(OC_2_H_5_)_4_, 98%) is an analytical reagent that was purchased from Aladdin. Fly ash was obtained from Tianchi Energy Power Plant of Xinjiang Tebian Electric Apparatus Stock Co., Ltd. (Xinjiang, China), with the main components being silicates and aluminates (75%). Cement was purchased from Bopo Run Refractory Material Co., Ltd. (Henan, China), with the main component being silicates (85%). All solutions were prepared using deionized water produced using an ultrapure water machine. Universal testing machine (MTS Corporation, Eden Prairie, MN, USA); UV-Visible-Near Infrared Spectrophotometer (Shimadzu Corporation, Kyushu, Japan).

### 2.2. Synthesis of Ginkgo Leaf Biochar Modified by TEOS

**Figure 2:**
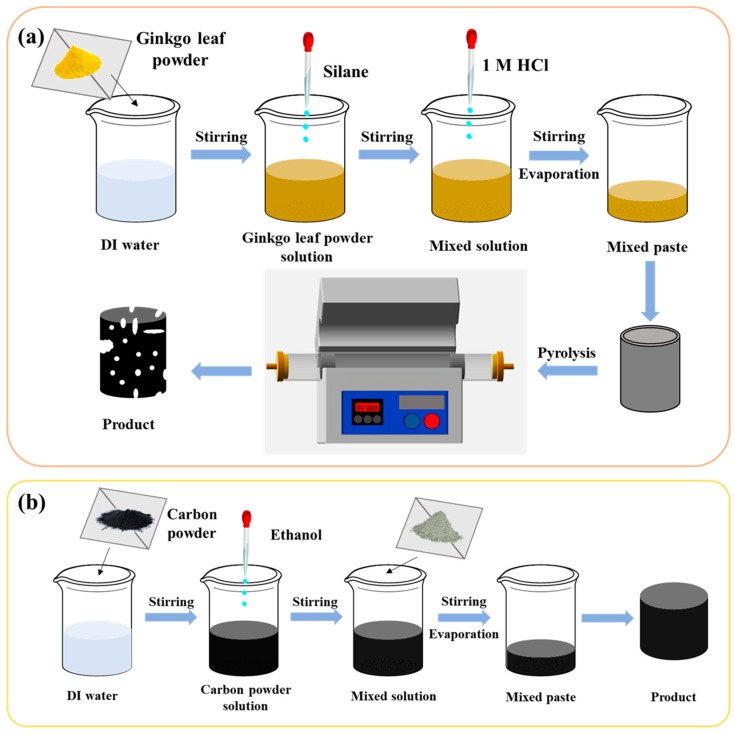
(**a**) Synthesis of ginkgo leaf biochar modified by TEOS; (**b**) inorganic-skeleton-reinforced bio-carbon with cement and fly ash.

As shown in Figure 2a, ginkgo leaf powder (GL, 2.5 g), water (10 mL), and ethanol (2.5 mL) were mixed by ultrasound for 5 min to make the mixture uniform, then different amounts of TEOS (0.5, 1, 1.5, 2, and 2.5 mL) were added, and then hydrochloric acid (1 mL) was dropped into the mixed solution and it was stirred for 2 h. Then, the mixed solution was heated in a water bath to 80 °C and kept there for 12–15 min (the magneton just leaks out) until it was mushy. The sample was placed into a porcelain boat for pyrolysis at 800 °C for 2 h with a heating rate of 5 °C/min under the condition of argon atmosphere. After cooling down to ambient temperature, the obtained sample was immersed into the HCl solution (1 mol·L^−1^) and stirred for 1 h, and then washed repeatedly with ultra-pure water until the pH value reached 7. Finally, the black sample was dried in a vacuum oven at 60 °C for 12 h and denoted as GL-TEOS-x (x = 0.5, 1, 1.5, 2, and 2.5). Because the optimal addition of TEOS is 1.5 mL, GL-TEOS is referred to herein as GL-TEOS-1.5 unless otherwise stated. As a contrast, the samples obtained from single ginkgo biloba leaf powder and silane with other conditions being kept constant are named as GL and Si, respectively. 

### 2.3. Synthesis of Inorganic-Skeleton-Reinforced Bio-Carbon with Cement and Fly Ash

As shown in Figure 2b, carbon powder (CP, 2 g), water (2 mL), and cement (0.4, 0.8, 1.2, 1.6, and 2.0 g) were stirred together for five minutes, followed by ultrasonic for five minutes, and then stirred for half an hour. After drying the mixture at 80 °C for 1 h, a small amount of water was added, and then it was dried at 80 °C for 1 h; this was repeated twice. Finally, the black sample was dried under a vacuum at 80 °C for 2 h. In the synthesis process, the hydration process of cement will form inorganic-gel-like polymers (IG), thus the obtained carbon materials are denoted as CP-IG-x (x = 0.4, 0.8, 1.2, 1.6, and 2.0).

The synthesis of inorganic-skeleton-reinforced bio-carbon with fly ash is the same as CP-IG-x, except for the addition of different ingredients: carbon powder (2 g), water (1.5 mL), 1 M KOH (1.5 mL), and fly ash (0.4, 0.8, 1.2, 1.6, and 2.0 g). Under alkaline conditions, fly ash is activated. It undergoes a reaction similar to the cement hydration process for crosslinked inorganic networks, ultimately forming geological polymer (GP). Therefore, the prepared materials are designated as CP-GP-x (x = 0.4, 0.8, 1.2, 1.6, and 2.0). 

### 2.4. Materials’ Characterization

X-ray diffraction (XRD) patterns of GL and GL-TEOS-x were obtained on an XRD analyzer (D8-Advance, Bruker AXS, Karlsruhe, Germany) equipped with a diffracted-beam monochromator using Cu Kα radiation (50 kV, 40 mA). Infrared analysis was performed using a Nicolet 170SX Fourier transform infrared (FTIR) spectrometer (Bruker corporation, Karlsruhe, Germany). The sample was scanned in the range of 400–4000 cm^−1^ after it was mixed with KBr and pressed at room temperature. The Raman spectra were obtained using Raman spectroscopy (Horiba Scientific, Paris, France) with a 532 nm blue laser beam. Using the Raman test results, the maximum values in the corresponding D-band and G-band regions were directly taken as their intensities. The surface area was tested by the Brunauer–Emmer–Teller (BET) method with the use of nitrogen adsorption/desorption measurement (V-Sorb 2800P, Anhui, China). All samples were degassed in a vacuum at 200 °C for 5 h before sorption experiments.

### 2.5. Pollutants’ Removal

At present, with the rapid development of the printing and dyeing industry, the environmental problems caused by the discharge of dye wastewater are increasingly prominent. Most of the dyes currently used (such as methylene blue, acid fuchsin, methyl violet, and so on) do not degrade easily because of their aromatic structure, causing serious harm to the water environment and human life and health. Methylene blue (MB) is a thiazide dye. Although it has a good effect in the treatment of methemoglobin, if the dose of MB used is excessive, it will cause diseases such as Heinz-body anemia and red blood cell morphological changes [35,36,37,38]. To investigate the absorption capacity of GL, GL-TEOS-x, CP, CP-IG-x, and CP-GP-x, MB was selected as the adsorbed substance. In detail, 5 mg of each of acetonitrile, GL, GL-TEOS-x, CP, CP-IG-x, and CP-GP-x were added into 5 mL of MB solution with an initial concentration range from 50 to 700 mg·L^−1^. This was followed by shaking the mixtures continually for 2 h. Each sample was passed through a Teflon filter to separate particles from the supernatant. Residual concentrations of MB in the filtrate were quantified by measuring the UV absorbance at 664.5 nm. The adsorption capacity can be calculated according to Equation (1).

(1) $$Q_{t}=\frac{\left(C_{0}-C_{t}\right)V}{m}$$

where Q_t_ (mg/g) is adsorption capacity of materials at different time intervals; C_0_ (mg/L) and C_t_ (mg/L) are the initial and residual concentrations of MB, respectively; V (L) is the volume of MB solution; and m (g) is the mass of the absorbent.

### 2.6. Compressive Strength Test

**Figure 3:**
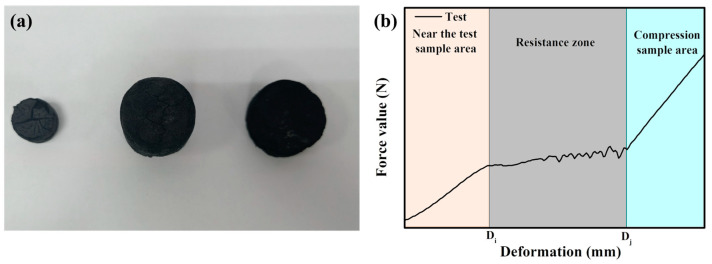
(**a**) Modified carbon material sample (from left to right are GL-TEOS, CP-IG, and CP-GP); (**b**) schematic diagram of a three-interval compressive strength measurement curve. Left: near the test sample area; middle: stress resistance zone; right: the area where the sample was crushed.

In order to facilitate the stress test, the material was prepared into cylindrical material, as shown in Figure 3a. The universal testing machine was applied to test the compressive strength of cylindrical materials, and the measuring curve of the real-time pressure measuring system was obtained. As shown in Figure 3b, it can be observed that the curve presents three different pressure displacement intervals: near the sample area, resistance zone, and compressed sample area. The authenticity and reliability of the mechanical property curve are verified by repeated experiments. Therefore, mathematical statistics can be used to scientifically fit the force value (F) of the sample. The method is as follows: adding the pressure values of each data point in the broken sample area of each curve and then dividing the total displacement in the broken sample area to obtain the average pressure of each curve. It is worth noting that all statistical pressures must belong to the area of the broken sample. The calculation is presented in Formula (2) as follows:

(2) $$F=\frac{\sum_{i=i}^{i=j}F_{i}}{D_{j}-D_{i}}$$

where *F_i_* and *F_j_* are the pressures at the beginning and end, respectively, of the broken sample area. At the same time, *D_i_* and *D_j_* are the distance to the start length and end length, respectively, of the broken sample area.

Then, Formula (3) is used to calculate the compressive strength:

(3) $$p=\frac{F}{S}$$

where *p* is the compressive strength and F is the calculated pressure. At the same time, S is the cross-sectional area of the cylindrical material.

## 3. Results and Discussions

### 3.1. Characterization of GL-TEOS

**Figure 4:**
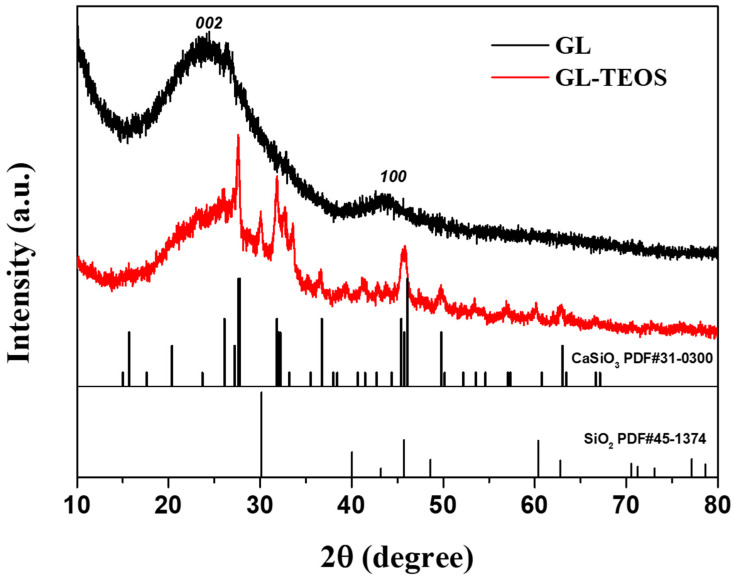
XRD patterns of GL and GL-TEOS.

The crystalline structures of GL and silane-modified GL were analyzed by X-ray diffraction (XRD). As shown in Figure 4, the diffraction peaks of GL at 2θ = 24° and 2θ = 43° belong to the (002) and (001) faces of graphite carbon, respectively [39,40,41,42]. The broad band of the (002) plane reveals that the biochar from ginkgo biloba leaves has a high graphitization degree. After adding TEOS, the intensity of the (002) peak decreased significantly, and the (100) peak almost disappeared, indicating that the graphitization degree of GL-TEOS decreased dramatically. In addition, for GL-TEOS, the characteristic peaks at 30.2°, 40.2°, 45.8°, and 60.5° are in correspondence with SiO_2_, and the characteristic peaks at 28.1°, 32.3°, and 45.1° matched well with CaSiO_4_, indicating that silicon enters GL-TEOS in the form of inorganic substances. 

**Figure 5:**
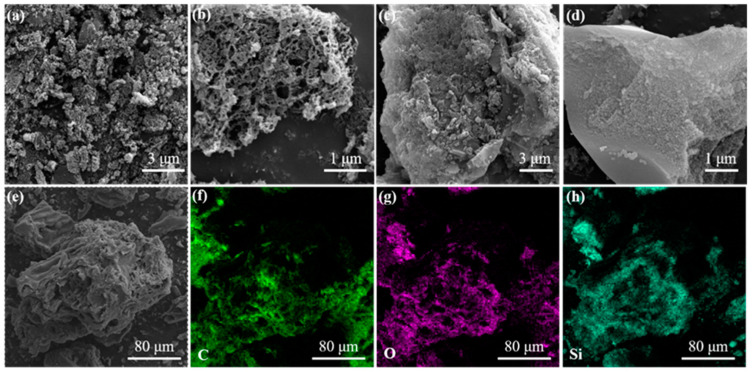
SEM images of (**a**,**b**) GL and (**c**–**e**) GL-TEOS, and the corresponding EDX mapping of (**f**) C, (**g**) O, and (**h**) Si.

The morphology and structure of GL and GL-TEOS were evaluated by SEM. As shown in Figure 5a,b, the pristine GL presents a sponge-like network form with a large number of continuous interconnected porous structures, which can be attributed to the evaporation of water and the acid erosion during pyrolysis. The cross-linked porous structure can provide channels for ion diffusion and transport. After silane modification (Figure 5c,d), GL-TEOS shows a smooth and dense surface structure with limited pores and channels. This may be due to the consumption of some HCl in the silane hydrolysis process, resulting in fewer pore formations and some inorganic components entering the material to fill the pores, thus significantly reducing the porosity of the material. The energy-dispersive X-ray spectroscopy (EDX) displays the existence of C, O, and Si signals, and the elements are evenly distributed on the surface of GL-TEOS, indicating that Si has successfully bound to the GL matrix (Figure 5e–h). 

**Figure 6:**
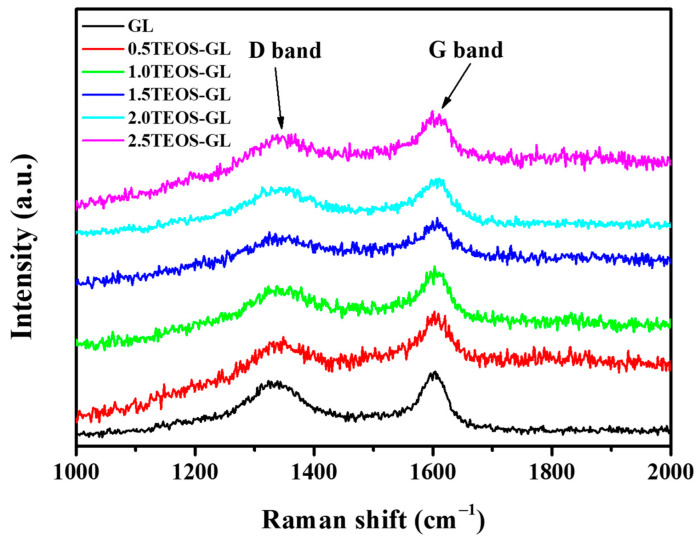
Raman spectra of GL and GL-TEOS-x (x = 0.5, 1, 1.5, 2, and 2.5).

The Raman spectra of GL and GL-TEOS-x present two characteristic peaks at 1350 cm^−1^ and 1600 cm^−1^, corresponding to the D-band and G-band, respectively (Figure 6). The D-band vibration originates from defects and disorder structures in carbon, while the G-band comes from the vibration of sp^2^ hybrid carbon in the microcrystalline structure. Accordingly, the D-band is related to the degree of crystal defects, while the G-band represents the microcrystalline structure, and the degree of defect and graphitization can be evaluated by the ratio of the D-band to G-band (I_D_/I_G_) [43,44,45,46]. After modification with TEOS, the I_D_/I_G_ ratios of GL-TEOS-x (ratios of x = 0.5, 1, 1.5, 2, and 2.5 assigned to 1.08, 1.18, 1.31, 1.20, and 1.25, respectively) are greater than that of pristine GL (0.8), and the maximum value of I_D_/I_G_ (1.31) can be obtained when the amount of TEOS is 1.5 mL. This indicates that the entry of silane decreases the graphitization degree of GL-TEOS-x. Moreover, from Table 1, it can be seen that the I_D_/I_G_ value is consistent with the change in the compressive strength and product quality. Specifically, with the increase in TEOS, the silicon content entering GL first increases and then tends to a stable value. The introduction of TEOS leads to an increase in the disorder of the material and a decrease in the graphitization degree, which is consistent with the XRD results. These results indicate that the improvement in the mechanical property of the material comes from the inorganic skeleton formed by the entry of silane. 

**Figure 7:**
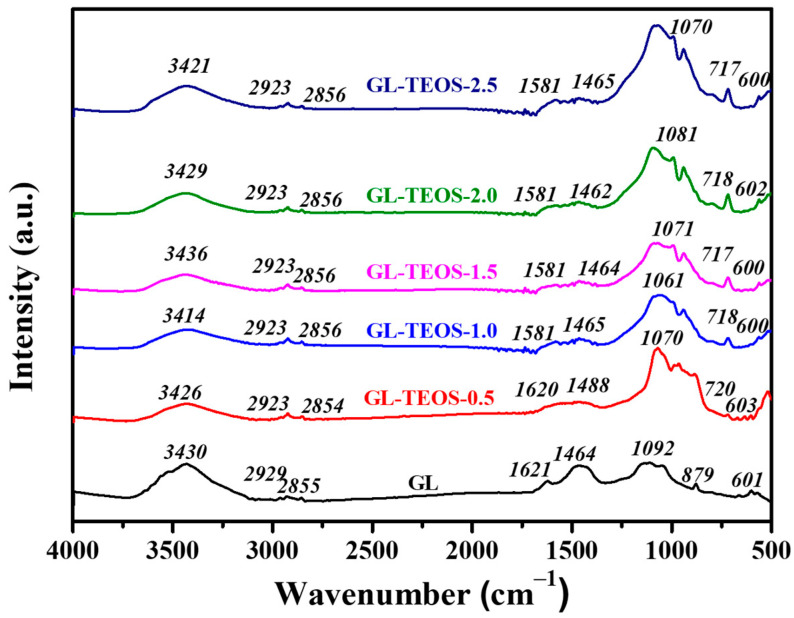
FT-IR spectra of GL and GL-TEOS-x (x = 0.5, 1, 1.5, 2, and 2.5).

Figure 7 shows the FT-IR spectra of silane-modified biochar with different silane contents. The strong and broad absorption band at about 3430 cm^−1^ is attributed to the O-H tensile vibration peak of the GL. It can be found that, compared with GL, the O-H peak of GL-TEOS-x exhibits a slight shift, which can be attributed to the introduction of silicon [47]. The absorption bands around 2929 cm^−1^ and 2855 cm^−1^ correspond to stretching vibrations of -CH_2_ and -CH_3_ groups on the surface, while the absorption bands of GL-TEOS-x show negligible changes, which could be due to the formation of silicon dioxide structures existing inside the material, rather than on the surface. The absorption bands near 1621 cm^−1^ and 1464 cm^−1^ correspond to the total aromatic absorption bands caused by the stretching of the C-C bond of the benzene ring [48,49]. The characteristic peaks at 1070 cm^−1^ and 717 cm^−1^ are assigned to Si-O-Si and Si-O structures, respectively. It is suggested that there is a silica network in GL-TEOS-x, which may be formed by the interaction between the phenolic hydroxyl group in biochar and the alcohol group of silane [50,51,52]. The silica network is conducive to promoting mechanical properties by providing a supporting skeleton. The absorption band near 600 cm^−1^ is assigned to the C-O tensile vibration peak on the surface, which is not affected by the introduction of silane, indicating that there is no reaction between Si and the C-O bond on the surface. Combined with all of the above results, this implies that the formation of the inorganic skeleton via silane hydrolysis and condensation is successful.

### 3.2. Mechanical Properties of Modified Carbon Materials

**Figure 8:**
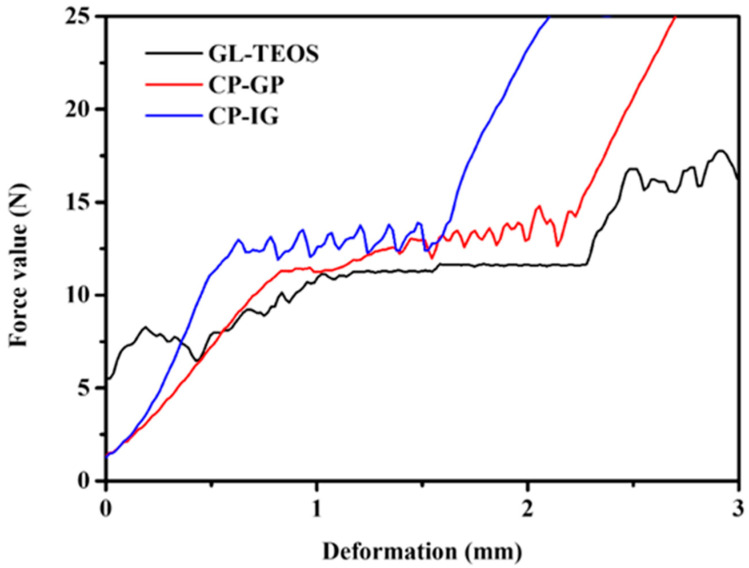
Compression test curve of GL-TEOS, CP-GP, and CP-IG.

In general, there is a limit to the increase or decrease in the compressive strength of modified materials, and the maximum improvement in compressive strength achieved by adding a modifying agent after the synthesis of carbon materials depends on the type of agent chosen. Figure 8 shows the test results corresponding to three materials, with the appearance of three corresponding regions. For the modified carbon materials that were prepared, the compressed sample area appears earlier, which may be because the fractured pieces are also stuck together. The corresponding calculated results are shown in Table 1, which indicate that the quality of silicon-modified biochar first increases and then tends toward a constant value with increasing amounts of silicon. The product quality of GL-TEOS-1.5 is 0.77 g, which is less than the sum of the product quality obtained by adding only 1.5 mL of TEOS and the product quality obtained by adding only ginkgo leaves. This may be because, during the thermal decomposition process of the carbon material, some of the silicon elements are carried away by volatile organic compounds with the airflow. TEOS was added during the preparation of ginkgo biloba leaf biochar, and the inorganic framework produced by the hydrolysis and condensation of silicon alkoxide significantly enhanced the mechanical properties of the biochar. The addition of TEOS changed the powder-like charcoal material that was originally obtained into a regular cylindrical shape. The maximum compressive strength of reinforced bio-carbon material can reach 88.9 kPa, thanks to the strong support provided by the silicon-oxygen network formed by silane alkoxide. For CP-IG-x, the compressive strength increases with the addition of cement. The maximum compressive strength of CP-IG-x can reach 124.6 kPa, which is related to the hydration reaction of cement to form a silicon-oxygen network. Specifically, after mixing with water, the mineral particles immediately undergo a chemical reaction with water and form hydration products, resulting in heat release and volume expansion, forming an inorganic skeleton. In this hydration process, the carbon powder will adhere to the inorganic skeleton, thereby enhancing the mechanical properties. As for CP-GP-x, in the synthesis process, the activation of fly ash will form geological polymer (GP) as an inorganic skeleton. The inorganic skeleton of CP-GP-x has a silicon-aluminum oxide structure, which is formed by the alternative of silicon tetrahedra and aluminum tetrahedra through sharing oxygen atoms. This skeleton provides a supporting effect for carbon materials, which is effective in improving the mechanical properties of carbon materials. Therefore, the mechanical properties of CP-GP-x are correlated to the amount of geopolymer, with a maximum value of 36.8 kPa. From the above results, it is shown that the compressive strength of carbon materials can be effectively improved by selecting appropriate modification methods and inorganic reinforcement agents.

### 3.3. Adsorption Performance of Modified Carbon Materials

**Figure 9:**
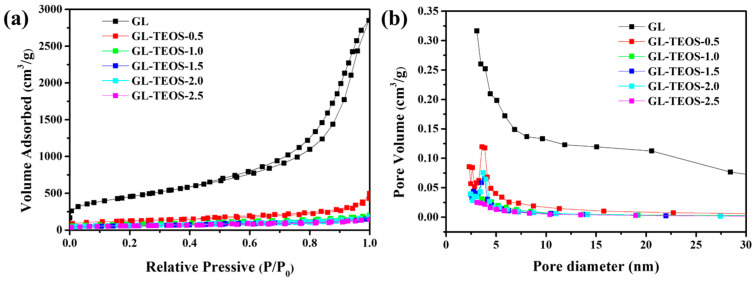
(**a**) Nitrogen adsorption/desorption isotherms and (**b**) corresponding pore size distribution of GL and GL-TEOS-x (x = 0.4, 0.8, 1.2, 1.6, and 2.0).

The pore structures of GL and GL-TEOS-x were characterized through nitrogen adsorption and desorption tests. Figure 9 shows the nitrogen adsorption and desorption isotherms and the corresponding pore structure distribution of GL and GL-TEOS-x (x = 0.4, 0.8, 1.2, 1.6, and 2.0). According to the IUPAC (International Union of Pure and Applied Chemistry) classification, the GL adsorption curve is a type IV isotherm adsorption curve, as shown in Figure 9a. At low pressures (P/P_0_ < 0.1), it has a high adsorption capacity, indicating that there are many micropores in GL. When the relative pressure (P/P_0_) is in the range of 0.4 to 1, a type H4 hysteresis loop can be observed, which is related to capillary condensation, indicating that GL contains a certain amount of mesopores. It is worth noting that the nitrogen adsorption and desorption curves of the silane-modified biochar exhibit the same type IV curves, but, with the increase in silane, the adsorption capacity of GL-TEOS-x decreases. At low pressures (P/P_0_ < 0.1), the high adsorption capacity indicates that there are micropores in GL-TEOS-x. However, with the increase in silane, the proportion and porosity of micropores significantly decreased, which is consistent with the SEM results. Specifically, the declining trend first increased with the increase in silane and then became constant, consistent with the change in product quality. As shown in Table 2, the specific surface area of GL is 1614 m^2^/g and the total pore volume is 4.41 cm^3^/g. After the addition of silane, the specific surface area decreased to 422 m^2^/g and the pore volume decreased to 0.77 cm^3^/g. This is because the introduction of silane changes the structure of GL-TEOS-x from porous and rough to strict and smooth, resulting in a decrease in pore volume. Further, with the increase in silane, the changes in pore volume and specific surface area are consistent with the changes in product quality, indicating that the addition of silane is the main reason for this change. Figure 9b presents the pore size distribution of GL and GL-TEOS-x (x = 0.5, 1, 1.5, 2, and 2.5). It can be seen that all materials have both micropores and mesopores. The proportion of micropores of GL is greater than that of mesopores. The number of micropores can provide a large number of active sites for ion storage, material adsorption, and surface reaction. However, GL-TEOS-x with a low apparent pore volume is dominated by micropores. The pore size distribution of GL-TEOS-x also changes with the addition of silane, which first decreases and then stabilizes. A higher pore volume and surface area can greatly enhance the adsorption performance of materials, but the addition of TEOS leads to a decrease in pore volume, and the adsorption performance may also slightly decline.

**Figure 10:**
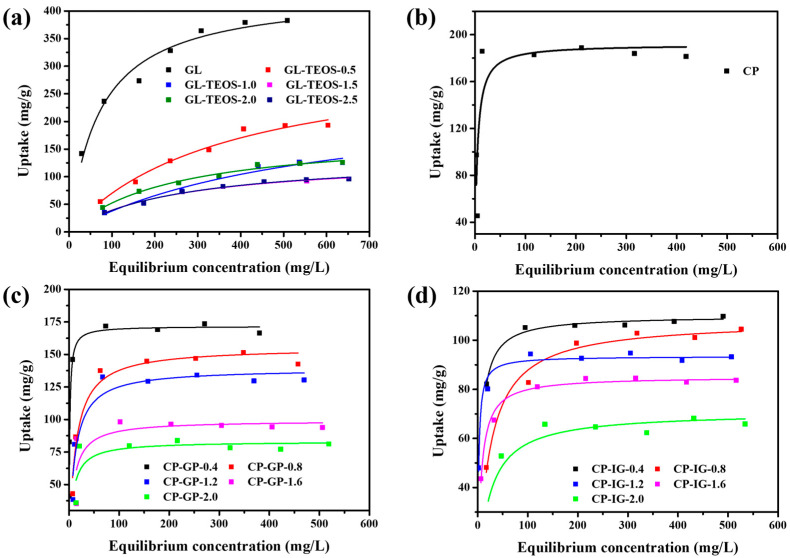
Equilibrium adsorption isotherms of MB on (**a**) GL and GL-TEOS-x (x =0.5, 1, 1.5, 2, and 2.5); (**b**) CP; (**c**) CP-GP-x (x = 0.4, 0.8, 1.2, 1.6, and 2.0); and (**d**) CP-IG-x (x = 0.4, 0.8, 1.2, 1.6, and 2.0).

The adsorption behavior of three modified carbon materials on the dye pollutants is evaluated by equilibrium adsorption isotherms. As shown in Figure 10 and Table 3, the three materials present different adsorption capacities. On the whole, the adsorption capacity of MB increases sharply in the relatively low concentration of MB solution. With the concentration increasing gradually, the adsorption capacity of MB tends to be saturated. It is obvious that GL exhibits the highest adsorption capacity (434 mg/g) for MB, which could be attributed to the high specific surface area and porosity of GL (Figure 10a, Table 3). However, the adsorption performances of GL-TEOS-x decrease with the increase in TEOS, and the decreasing trend is consistent with the changes in product quality, indicating that this attenuation is caused by the decrease in particle size, surface smoothness, pore volume, and specific surface area of the material after the addition of non-organic components. In addition, the changes in the adsorption performances of CP-GP-x and CP-IG-x are different from that of GL-TEOS-x. Specifically, the decreasing trend of adsorption capacity after silane addition is direct, but the change with the increase in silane is slight. The adsorption performances of CP-GP-x and CP-IG-x vary greatly with the number of additives. For pristine CP, the maximum MB adsorption capacity is 194.4 mg/g (Figure 10b and Table 3). After the addition of geological polymer, the adsorption performance of CP-GP-x slightly decreases from 171.7 to 93.2 mg/g (Figure 10c and Table 3). Although the performance has decreased, it is acceptable compared with the significant improvement in mechanical properties. The adsorption performances of CP-IG-x with the addition of geopolymer demonstrate a different trend from those of CP-GP-x. The adsorption capacity of carbon material is reduced twofold after the addition of IG, but the subsequent change is relatively small (Figure 10d and Table 3). This may be because of the more compact structure of the geopolymer, resulting in a smaller pore volume. It is worth noting that, although the adsorption performance has decreased, the material can be formed into blocks owing to the improved mechanical properties. This is beneficial for the collection and reuse of the material after simple filtration, effectively improving the utilization efficiency.

## 4. Conclusions

In summary, to improve the poor mechanical properties at the macroscopic level of bio-based materials, an inorganic skeleton reinforcement strategy was proposed and developed. It was implemented through two approaches as follows: (1) the addition of silane to the synthesis process of bio-based carbon materials to generate an inorganic framework in situ; and (2) the mixing of pre-synthetic bio-based carbon materials with a precursor that can form an inorganic framework, enabling the bio-based carbon materials to adhere to the generated inorganic framework. The formation of the inorganic frameworks is verified by systematic characterization of the chemical composition and microstructure of the as-prepared materials. Although the test results indicate that a decent improvement in mechanical properties comes at the expense of adsorption performance, the modified carbon materials with enhanced compressive properties can be molded into blocks, which facilitates filtering, collection, and reutilization, effectively increasing the utilization efficiency. In addition, the improved reusability of these materials is conducive to achieving the balance between mechanical and physio-chemical properties. This research provides new insights for improving the mechanical properties of bio-based carbon materials, thus encouraging more research to expand the practical applications.

## Figures and Tables

**Table 1:** Comparison of the product quantity, theoretical carbon content, and compressive strength of different carbon materials.

Material	Product Quantity ^1^ (g)	Carbon Content (%)	Compressive Strength (kPa)
GL	0.25	100	0
GL-TEOS-0.5	0.48	55.6	14.3
GL-TEOS-1.0	0.62	36.2	45.3
GL-TEOS-1.5	0.77	30.5	88.9
GL-TEOS-2.0	0.84	32.9	74.9
GL-TEOS-2.5	1.06	31.6	79.8
Si	0.58	0	-
CP	2	100	-
CP-GP-0.4	2.4	83.3	12.3
CP-GP-0.8	2.8	71.4	18.5
CP-GP-1.2	3.2	62.5	24.3
CP-GP-1.6	3.6	55.5	30.3
CP-GP-2.0	4.0	50	36.8
CP-IG-0.4	2.4	83.3	8.8
CP-IG-0.8	2.8	71.4	13.9
CP-IG-1.2	3.2	62.5	34.9
CP-IG-1.6	3.6	55.5	65.4
CP-IG-2.0	4.0	50	124.6

^1^ The product quantity in Table 1 refers to the specific weight of the prepared product.

**Table 2:** The specific surface areas investigated by *Brunauer–Emmett–Teller* (BET) (S_BET_) and pore volume of GL and GL-TEOS-x (x = 0.5, 1, 1.5, 2, and 2.5).

Material	S_BET_ (m^2^·g^−1^)	Pore Volume (cm^3^·g^−1^)
GL	1614	4.41
GL-TEOS-0.5	422	0.77
GL-TEOS-1.0	270	0.31
GL-TEOS-1.5	197	0.25
GL-TEOS-2.0	213	0.28
GL-TEOS-2.5	201	0.26

**Table 3:** The maximum adsorption capacity of the sample of GL and GL-TEOS-x (x = 0.5, 1, 1.5, 2, and 2.5); CP; CP-GP-x (x = 0.4, 0.8, 1.2, 1.6, and 2.0); and IP-CP-x (x = 0.4, 0.8, 1.2, 1.6, and 2.0) to MB.

Material	Adsorption Capacity (mg/g)	Material	Adsorption Capacity (mg/g)	Material	Adsorption Capacity (mg/g)
GL	434	CP	194.4	CP	194.4
GL-TEOS-0.5	325	CP-GP-0.4	171.7	CP-IG-0.4	109.9
GL-TEOS-1.0	251	CP-GP-0.8	155.4	CP-IG-0.8	100.8
GL-TEOS-1.5	134	CP-GP-1.2	139.0	CP-IG-1.2	93.4
GL-TEOS-2.0	177	CP-GP-1.6	99.1	CP-IG-1.6	85.2
GL-TEOS-2.5	136	CP-GP-2.0	93.2	CP-IG-2.0	70.8

## Data Availability

The data presented in this study are available upon request from the corresponding author.
